# Dual exon skipping in myostatin and dystrophin for Duchenne muscular dystrophy

**DOI:** 10.1186/1755-8794-4-36

**Published:** 2011-04-20

**Authors:** Dwi U Kemaladewi, Willem MH Hoogaars, Sandra H van Heiningen, Samuel Terlouw, David JJ de Gorter, Johan T den Dunnen, Gert Jan B van Ommen, Annemieke Aartsma-Rus, Peter ten Dijke, Peter AC 't Hoen

**Affiliations:** 1 Center for Human and Clinical Genetics, Leiden University Medical Center, Postzone S4-P, PO Box 9600, Leiden, 2300RC, the Netherlands; 2 Department of Molecular and Cell Biology, Leiden University Medical Center, Postzone S1-P, PO Box 9600, Leiden, 2300RC, the Netherlands; 3 Institute for Molecular Cell Biology, University of Münster, Schlossplatz 5, Münster, D-48149, Germany

## Abstract

**Background:**

Myostatin is a potent muscle growth inhibitor that belongs to the Transforming Growth Factor-β (TGF-β) family. Mutations leading to non functional myostatin have been associated with hypermuscularity in several organisms. By contrast, Duchenne muscular dystrophy (DMD) is characterized by a loss of muscle fibers and impaired regeneration. In this study, we aim to knockdown myostatin by means of exon skipping, a technique which has been successfully applied to reframe the genetic defect of dystrophin gene in DMD patients.

**Methods:**

We targeted myostatin exon 2 using antisense oligonucleotides (AON) in healthy and DMD-derived myotubes cultures. We assessed the exon skipping level, transcriptional expression of myostatin and its target genes, and combined myostatin and several dystrophin AONs. These AONs were also applied in the *mdx *mice models via intramuscular injections.

**Results:**

Myostatin AON induced exon 2 skipping in cell cultures and to a lower extent in the *mdx *mice. It was accompanied by decrease in myostatin mRNA and enhanced *MYOG *and *MYF5 *expression. Furthermore, combination of myostatin and dystrophin AONs induced simultaneous skipping of both genes.

**Conclusions:**

We conclude that two AONs can be used to target two different genes, *MSTN *and *DMD*, in a straightforward manner. Targeting multiple ligands of TGF-beta family will be more promising as adjuvant therapies for DMD.

## Background

Duchenne muscular dystrophy (DMD) is an X-linked recessive neuromuscular disorder, which is caused by dystrophin deficiency in muscle fibers. DMD fibers are more sensitive to muscle damage, leading to degeneration and replacement of muscle fibers by fat and connective tissue (fibrosis). Monaco *et al *found that frame shift mutations in the *DMD *gene will lead to a truncated and non-functional form of dystrophin [1], which become the primary cause of the disease. However, mutations which maintain the *DMD *open reading frame result in shorter dystrophin proteins that retain the essential actin binding-, cysteine rich- and carboxy terminal domains, and thus are partly functional [1]. Patients with such mutations develop the less severe Becker muscular dystrophy. This reading frame rule holds true for ~91% of DMD cases [2] and has inspired the development of the exon skipping strategy, which employs antisense oligonucleotides (AONs). These small synthetic RNA molecules are complimentary to exonic or splice site sequences, thereby upon hybridization are able to modulate exon inclusion by the splicing machinery (recently reviewed in [3-5]).

Comprehensive studies done by our group and others have provided the proof of principle of the therapeutic feasibility of the AON to reframe dystrophin transcripts and restore dystrophin synthesis, both *in vitro*[6-8] and *in vivo *using the *mdx *and hDMD mice[9-11]. Subsequent clinical trials have shown that two different AON chemistries, either 2'-*O*-methyl phosphorothioate (2'OMePS)[12] or phosphorodiamidate morpholino oligomer (PMO)[13] targeting *DMD *exon 51 can restore local dystrophin synthesis in DMD patients with no to minimum side effect.

However, other major facets of DMD pathology which include severe muscle wasting, fibrosis and flawed muscle regeneration may reduce the efficacy of the *DMD *exon skipping therapy. In addition, as DMD patients suffer from muscle degeneration from their early life, myoblasts undergo extensive division in an attempt to regenerate, which eventually leads to exhaustion of the muscle regenerative potential [14-16]. Therefore, several additional therapies have been considered to overcome these problems, in which myostatin inhibition has received considerable interest.

Myostatin or Growth and Differentiation Factor-8 (GDF-8), an evolutionary conserved TGF-β family member that is expressed predominantly in skeletal muscle, is a potent muscle growth inhibitor. Natural mutations or targeted knockdown in the *MSTN *genes, which lead to non-functional proteins are associated with hypermuscularity in several organisms such as mice, cattles, dog, horse, sheep and human [17-21]. Myostatin signaling requires binding to the Activin type IIB receptor (AcvRIIb) and Activin receptor-like kinase 4/5 (ALK4/5) receptor complex and subsequent phosphorylation of downstream Smad2 and Smad3 [22], which further influences myogenesis through transcriptional regulation of cell cycle and myogenic regulatory factors [23-27]. Several strategies to block myostatin such as neutralizing antibodies [28-30], overexpression of its inhibitory propeptide [31-34], or administration of soluble AcvRIIB [35] induced increases in muscle mass and force and reduced fibrosis in the *mdx *mice [36].

A recent study by Dumonceaux *et al *[37] targeted the myostatin receptor AcvRIIb using shRNA and coupled it with a modified U7 small nuclear RNA to restore dystrophin in single adeno-associated vectors (AAV). This study showed that combination therapy has an added value in improving muscle physiology. However, as recently reviewed by Tang *et al *[38], one major limitation of AAV-based gene therapy approach is the immune response against the vector and/or transgene.

**Figure 1:**
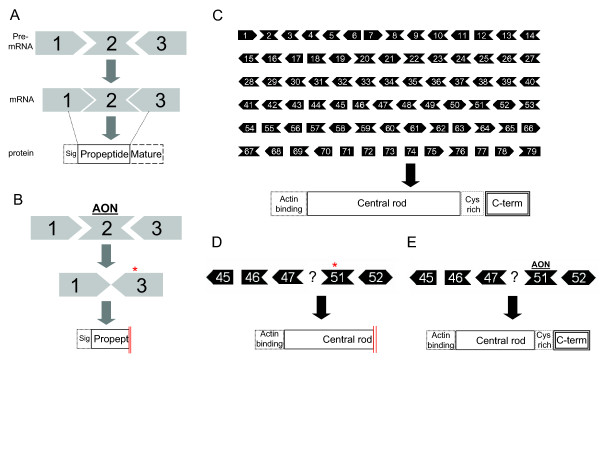
**Schematic overview of myostatin and dystrophin exon skipping**. The myostatin gene (*MSTN*) consists of three exons, whereas the protein consists of three domains: signaling (sig.), propeptide and mature domains. The position of each exon relative to each domain is denoted by broken lines (A). Antisense oligonucleotides (AON) targeting exon 2 will hybridize and hide the exon from the splicing machinery, resulting in skipping exon 2 upon mRNA splicing. The removal of exon 2 will disrupt the open reading frame (ORF; *) and the protein will lack part of the propeptide and the entire mature domain (B). Dystrophin gene (*DMD*) consists of 79 exons, whereas the proteins consist of actin binding-, central rod, cysteine rich- and C-terminal domains (C). One of the examples of DMD is deletion in exon 48-50 which disrupted the reading frame and introduces premature stop codon (*). Due to this mutation, part of the central rod and the entire Cys-rich and C-terminal domains are missing (D). In the therapy (currently in clinical trials), AON is directed towards exon 51. With the similar principle, exon 51 will be skipped upon mRNA splicing which in turn restores the ORF. This internally-deleted *DMD *will be translated as dystrophin with shorter central rod domain. However, since the essential C-terminal domain is retained, the protein is partially functional (E).

In this study we chose to use 2'OMePS AON to target *MSTN *exon 2 and disrupt its translational reading frame in order to knockdown its expression level. As it harbors the same chemistry modification as the PRO051 DMD AON currently in trial, we hypothesized that the administration of the two AONs as a cocktail would be achievable and may thus yield a combination treatment by simultaneously correcting the *Dmd *transcript and downregulating the *Mstn *transcript. In addition, as recently reviewed in [39], the 2'OMePS modification enhances the stability and increases the *in vivo *half-life compared to the previously studied butanol-tagged antisense [40] or siRNA-mediated RNA interference approaches targeting myostatin [41]. We evaluated its feasibility to induce exon skipping and downregulate *MSTN *expression in myotubes cultures and DMD mouse models. Furthermore, we combined it with several DMD AONs to look into the possibility of skipping two genes simultaneously (Figure 1).

## Methods

### Antisense oligonucleotides

AON with phosphorothioate backbones and 2'-*O*-methyl ribose modifications used in this study were synthesized and high-pressure liquid chromatography purified by *Eurogentec, Belgium*. The sequences are listed in Table 1.

**Table 1:** Antisense oligonucleotides used in this study

AON	Targeting	Sequences (5'-3')
AON1	Myostatin exon 2	guuugaugagucucaggauu
AON2	Myostatin exon 2	gccaaauaccagugccu
AON3	Myostatin exon 2	agccaauuuugcaacacugu
h8AON3	Human dystrophin exon 8	guacauuaagauggacuuc
h44AON1	Human dystrophin exon 44	cgccgccauuucucaacag
h51AON1	Human dystrophin exon 51	ucaaggaagauggcauuucu
h54AON1	Human dystrophin exon 54	uacauuugucugccacugg
M23D (+02-18)	Mouse dystrophin exon 23	ggccaaaccucggcuuaccu

### Cell culture

Human primary myoblasts obtained from a healthy donor (KM109) [42], immortalized myoblast cell line (7304-1) generated by expressing telomerase (hTERT) and cyclin-dependent kinase 4 [43], and DMD patient derived myoblasts with a deletion in exon 51-55 (DL589.2) [7] were used in this study. All of the cells mentioned above were seeded on thin layer collagen (1:30, PureCol, *Inamed Biomaterials, Fremont*). The primary cells were grown in Nut.Mix F-10 (HAM) supplemented with GlutaMax-I, 20% Fetal Bovine Serum (FBS) and 1% Penicillin/Streptomycin (*Gibco-BRL*) at 37°C, 5% CO_2_. The 7304-1 cells were cultured in Skeletal Muscle Cell Basal Medium supplemented with 15% FBS, 5 μg hEGF, 0.5 μg hFGF, 25 mg Fetuin, 5 mg Insulin and 200 μg Dexamethasone (PromoCell GmbH, Germany). 80-90% confluent culture was induced to differentiate into myotubes in DMEM supplemented with 2% FBS, 1% P/S, 2% Glutamax and 1% glucose (*Gibco-BRL*), except the 7304-1 in which 2% of Horse Serum was added instead of FBS.

### AON transfections

Myotubes were transfected with different dilutions of AON in 0.15 M NaCl using 2.5 μl polyethylenimine (ExGen 500, *MBI Fermentas*) per μg of AON for 3 hours.

### Animals and AON injections

All experiments were performed with 5-6 weeks old *mdx *mice under the approval of the Animal Experimental Committee (DEC07195) of the LUMC. In the single injection experiments, the AONs were administered into the gastrocnemius muscle at the dose of 40 μg per injection in 50 μl physiological salt. Control DMD AON (M23D) was injected in the contralateral muscle. After 4 days, the mice (n = 2) were sacrificed and muscles were isolated, snap frozen and sectioned at three different positions (proximal, medial and distal relative to the tendon) for total RNA isolation.

In the consecutive injections experiments, cocktails of MSTN or control AONs with M23 DMD AON were injected at the dose of 40+40 μg per injection into the gastrocnemius muscle. Four consecutive injections were performed with a 48 hours resting time between the second and third injection. The mice (n = 6) were sacrificed at 6 hours, 1 and 2 days after the last injection. The muscles were isolated, snap frozen and sectioned. Pool of sections from different area of the muscles was collected for RNA isolation.

### RNA Isolation, RT-PCR and Quantitative PCR analysis

Cell lysates were prepared using lysis buffer provided in the NucleoSpin RNA II kit (*Macherey-Nagel, Germany*) and RNA was isolated using the same kit according to manufacturer's instructions. Sectioned muscles were initially lysed using the same buffer and crushed with MagNA Lyser Green beads in the MagNA Lyser instrument (*Roche Diagnostics, Germany*) at 7000 oscillation speed for 2 × 20 seconds. RNA was isolated using the same procedure. The RNA quantity and integrity were measured using RNA 6000 Nanochip in the Agilent 2100 bioanalyzer (*Agilent Technologies*). cDNA was synthesized from 500 ng of RNA using RevertAid H Minus M-MuLV Reverse Transcriptase (*MBI Fermentas*) with 40 ng of random hexamer primer, according to the manufacturer's instructions. 10× diluted cDNA was then amplified by PCR using primers listed in Table 2. Myostatin amplifications were performed at 94°C (30 s), 56°C (30 s), 72°C (60 s) for 30 cycles. Dystrophin PCRs were performed in two rounds. The first PCR was performed by 20 cycles of 94°C (40 s), 60°C (40 s), 72°C (80 s). 1.5 μl of these reactions were then reamplified in nested PCRs by 32 cycles of 94°C (40 s), 60°C (40 s), 72°C (60 s). Quantitative PCRs were carried out in a 384-wells plate with 2 μl of 10X diluted cDNA, 1 μl of 1 pmol/μl forward primers, 1 μl of 1 pmol/μl reverse primers and 6 μl of iQ™ SYBR^® ^Green Supermix (*Bio-Rad*) in LightCycler 480 (*Roche Diagnostics, Germany*). Each measurement was performed in triplicates. Expressions of the genes of interest were normalized to housekeeping gene *GAPDH *and analyzed using ΔΔCt method with Gene Expression Analysis for iCycler iQ^® ^Real-Time PCR Detection System software developed by *Bio-Rad*. The expression values of 3-4 biological experiments were averaged. For the statistical analysis, the expressions in *MSTN *AON-transfected samples were compared to 500 nM control AON transfected samples in Student's t-test. P-values < 0.05 were considered significant.

**Table 2:** Primers used in this study

Primers	Sequences	Used for
Mstn Ex1F	GGAAACAGCTCCTAACATCAG	Myostatin exon 2 skip PCR
Mstn Ex3R	CTGAGCAGTAATTGGCCTTATATC	
Mstn Ex1F1	GATGACGATTATCACGCTAC	Myostatin QPCR
Mstn Ex2R1	GCACAAACACTGTTGTAGGA	
Myog Fw	GCCAGACTATCCCCTTCCTC	Myogenin QPCR
Myog Rev	AGGGATGCCCTCTCCTCTAA	
Myf5 Fw	CCACCTCCAACTGCTCTGAT	Myf5 QPCR
Myf5 Rev	GCAATCCAAGCTGGATAAGG	
Gapdh Fw	CAATGACCCCTTCATTGACC	Gapdh QPCR
Gapdh Rev	GACAAGCTTCCCGTTCTCAG	
DMD Ex6F	CTGGCTTTGAATGCTCTCATC	DMD exon 8 skip PCR
DMD Ex11R	GCTGTCAAATCCATCATGTACC	
DMD Ex42F	GTGATGACTGAAGACATGCC	DMD Exon 44 skip PCR
DMD Ex45R	TCTGTCTGACAGCTGTTTGC	
DMD Ex53F	TTCAGAATCAGTGGGATGAAG	DMD Exon 54 skip PCR
DMD Ex56R	CGTCTTTGTAACAGGACTGC	

## Results

### Myostatin AON induces exon 2 skip *in vitro*

Based on previously described guidelines [44], we designed three AONs targeting different regions in *MSTN *exon 2 gene and used previously designed AONs targeting *DMD *exons as controls (Table 1). All AONs consisted of 2'-*O*-methyl RNA and had a phosphorothioate backbone in order to resist endonucleases and RNaseH degradation. We evaluated their efficiency using immortalized human myoblasts (7304-1) as well as primary myoblasts from a healthy individual (KM109) and a DMD patient with a deletion in exon 51-55 (DL589.2). We used differentiated myotube cultures because the expression levels of myostatin and dystrophin were found to be higher in the differentiation than in proliferation stage (data not shown).

**Figure 2:**
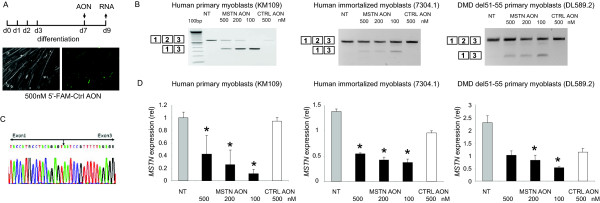
**Myostatin exon 2 skip in several myotubes cultures**. Human primary control (KM109) and DMD patient derived- (DL589.2) myoblasts were differentiated for 7 days before transfection with MSTN AON. Immortalized control (7304.1) myoblasts were differentiated for 2-3 days. A non-targeting, fluorescently-labeled AONs were transfected as control. Fluorescent nuclei were observed three hours post-transfection (A). RNA was isolated 2 days post-transfection. cDNA was synthesized using random hexamer (N6) primers and subjected for PCR using primers in exon 1 and 3 (B). Note the inverse dose-dependent skips in KM109 samples. Skip fragment was confirmed by sequencing analysis (C). Quantitative real-time PCR was performed using primers in *MSTN *exon 1 and 2, thereby depicting the expression of remaining full length or non-skipped transcript (D). Data are means ± SD from 3 to 4 independent experiments. Expression was normalized with GAPDH. Statistical analysis was performed using Student's t-test, using the 500 nM control AON-transfected samples as reference. *P < 0.05

To examine the feasibility to induce myostatin exon 2 skipping, different concentrations of each AON were transfected into the myotube cultures using the cationic polymer polyethylenimine (PEI). More than 80% of the cells showed specific nuclear uptake upon transfection with 5'-fluorescein (FAM)-labeled control AON (Figure 2A). RT-PCR performed two days post transfection (Figure 2B) and subsequent sequencing analysis (Figure 2C) showed the exclusion of exon 2 from the myostatin transcript in the myostatin AON-transfected cells, resulting in a premature stop codon formation. This internally truncated fragment was not observed in any of the non-transfected and control AON-transfected myotubes. One myostatin AON, namely AON1, gave the most consistent and highest skipping efficiency [Additional file 1]. Thus we further used the AON1 (addressed as myostatin AON from now on) and confirmed its exon skipping ability in human and to a lower extent in mouse cells models, using its perfect complementary to the human and mouse *MSTN *sequences (Figure 2B and not shown).

### AON-mediated exon skipping decreased myostatin transcript expression level and increased expression of myogenic regulatory factors

Next, we determined the expression level of *MSTN *transcript upon exon skipping by quantitative real time PCR. We used primers in exon 1 and exon 2, thereby detecting only the non-skipped products. As shown in Figure 2D, the levels of full length *MSTN *transcript decreased significantly in the myostatin AON-transfected samples, with slight variations in the knockdown levels between different cells. Notably, these decreases were achieved in an inverse dose-dependent manner, meaning that the most pronounced knockdown was achieved with 100 nM AON, which was the lowest concentration of myostatin AON tested. This effect was seen especially in the KM109 cells. In addition, the control AON induced less pronounced but still significant decreases of the full-length *MSTN *levels in some cells (7304-1 and DL589.2) (Figure 2D), which might be due to the transfection. Therefore, the expression levels in myostatin AON-transfected samples were compared with those in the control AON-transfected samples.

**Figure 3:**
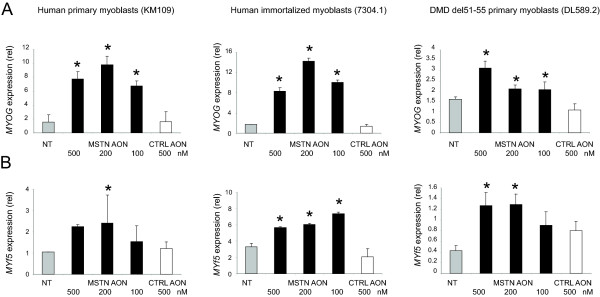
**The expression myogenic regulatory factors *MYOG *and *MYF5 *upon exon 2 skipping**. Cells were fused and transfected with different concentrations of AON as described in Figure 2. Total RNA was isolated and N6-primed cDNA was subjected to quantitative real-time PCR for *MYOG *(A) and *MYF5 *(B). Data are means ± SD from 3 to 4 independent experiments. Expression was normalized with GAPDH. Statistical analysis was performed using Student's t-test, using the 500 nM control AON-transfected samples as reference. *P < 0.05

As outlined in Figure 1B, myostatin skipping results in premature stop codon in exon 3 and thus non-functional myostatin protein. Several studies have described that myogenic cells respond to myostatin by down-regulating the expression of key transcriptional regulators of muscle development such as Pax3, Pax7, p21, MyoD, Myf5 and Myog [23-27,45,46], explaining its inhibitory effects in differentiation. Therefore, inhibition of myostatin should lead to an increased expression of these myogenic regulators and thus can be used as a functional readout of the myostatin knockdown. Correspondingly, knockdown of myostatin in differentiated myotubes cultures resulted in a consistent upregulation of *MYF5 *and *MYOG *expression (Figure 3A and 3B). The fold increase varied between 3-12 fold and dependent on the myoblast cultures used, which may reflect the different myogenic differentiation potential of the different myoblast cultures.

### Dual exon skipping in *MSTN *and *DMD *in control and DMD patient derived myotubes

**Figure 4:**
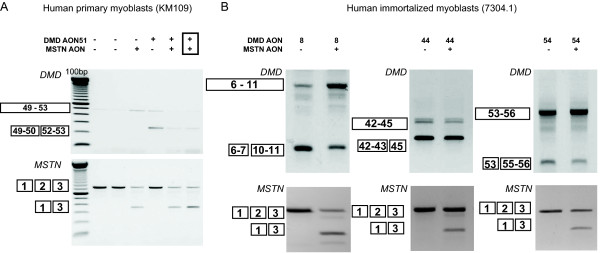
**Dual exon skipping of myostatin and dystrophin in control cells**. KM109 (A) and 7304-1 (B) myotubes were transfected with 200 nM of myostatin AON and AON targeting different *DMD *exons, namely exon 8, 44 and 54. The AONs were premixed (boxed) before complexing with the transfection reagent, or directly complexed (not boxed). RNA was isolated two days post-transfection and analyzed for myostatin or dystrophin skips by RT-PCR.

Our ultimate goal was to use AONs to simultaneously reframe the mutation in the *DMD *gene and to downregulate *MSTN *expression in order to correct the primary genetic defect as well as to enhance muscle regeneration. Therefore, we combined the myostatin AON with several DMD AONs used in the previous studies [6,7,47]. We specifically applied DMD AONs covering the hotspot mutated regions in *DMD *gene, namely h8AON3, h44AON1, h51AON1 and h54AON1 (Table 1) in control myotubes cultures (primary (KM109) or immortalized myoblasts (7304-1)). Myotubes were transfected with either myostatin or DMD AONs or a mix of both. As shown in Figure 4, *MSTN *exon skip was observed in all myostatin AON-transfected samples, regardless of the presence of DMD AON. Vice versa, all DMD AON-transfected myotubes showed the exon-specific dystrophin skips described before [6,7,47], regardless of the presence of MSTN AON. To investigate the possibility that the two AONs hybridized to each other rather than to their intended target, we varied the transfection conditions by mixing the AON before complexing with PEI, or preparing the AON-PEI complexes separately. However, no difference in their efficiency to induce skipping was found. We observed skipping with the total AON concentration of 200 nM (Figure 4) as well as 100 nM (not shown). These results demonstrated the feasibility of simultaneous antisense-mediated skipping of exons of two different genes.

**Figure 5:**
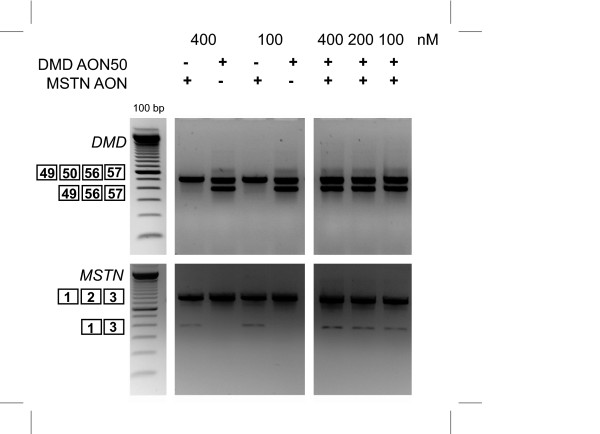
**Dual exon skipping of myostatin and dystrophin in DL589.2 DMD patient cells**. DL589.2 myotubes were transfected with 200 nM of myostatin AON and h50AON1 DMD AON. RNA was isolated two days post-transfection and analyzed for myostatin and dystrophin skips by RT-PCR.

To further assess its therapeutic potential, we performed dual exon skipping in the DL589.2 cells, which were derived from a DMD patient with an exon 51-55 deletion. Our previous study has shown that the reading frame can be corrected by an exon 50 skip upon transfection with h50AON1 [7]. As shown in Figure 5, combining this AON with the myostatin AON showed clear targeted skipping of both *DMD *and *MSTN*.

### Myostatin exon skipping *in vivo*

**Figure 6:**
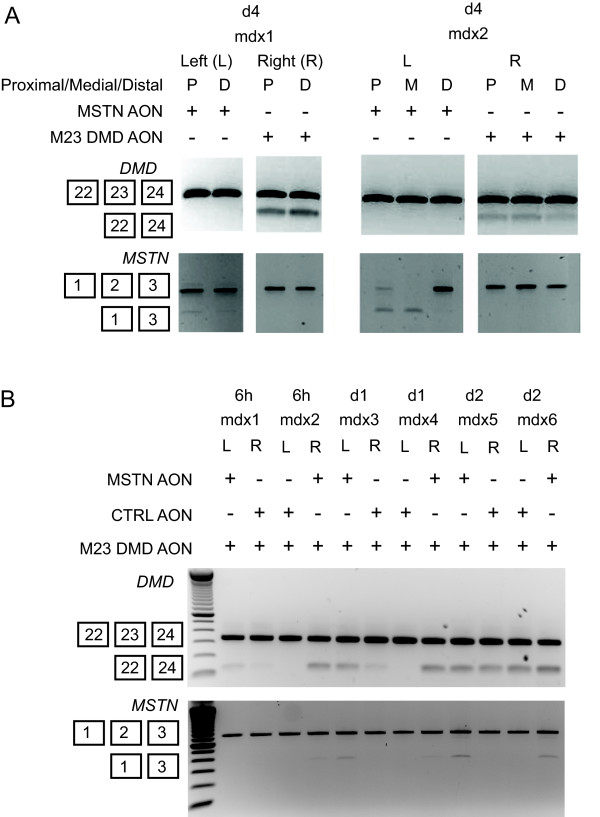
**Administration of myostatin AON in *mdx *mice**. Single dose of 40 μg MSTN AON was injected into the gastrocnemius muscles of *mdx *mice. Control M23 DMD AON were injected in the contralateral muscles. The animals were sacrificed 4 days after injection. RNA was isolated from three different parts of the muscles relative to the tendon: proximal (P), medial (M) and distal (E). RT-PCR analysis was performed to detect dystrophin (upper) or myostatin (lower) skips (A). Four times consecutive injections were performed with cocktails of AON containing 40 μg of m23 DMD AON and 40 μg of either myostatin or control AON into the gastrocnemius muscles of *mdx *mice. Injections were varied between contralateral muscles. The mice were sacrificed at 6 hours, 1 day and 2 days after the last injection. RNA was isolated from multiple areas within the muscles and analyzed for dystrophin (upper) or myostatin (lower) skips by RT-PCR (B).

We subsequently examined the ability of myostatin AON to induce exon skipping in the *mdx *mouse model. Myostatin AON was injected at a dose of 40 μg/injection into the gastrocnemius muscle of the *mdx *mice. DMD AON targeting exon 23 (M23D, previously denoted as M23D(+02-18)[9]) was injected into the contralateral muscle. This AON served as a positive control and was shown by many different groups including ours [9,11] to induce efficient and robust exon skipping. The mice were sacrificed at four days after injection. We classified different sections of the muscles as the proximal (P), medial (M) and distal (D) relative to the tendon and observed heterogenous patterns of *Mstn *exon 2 skips (Figure 6A). The skipping levels in the proximal and medial parts of the muscles were higher than in the distal, while *Dmd *skips appeared to be heterogenous throughout the muscles.

Our previous study in dystrophin exon skipping had indicated that higher skipping efficiencies could be obtained by multiple injections of the AON. In order to increase the efficiency of the myostatin AON and to answer whether dual skipping can be achieved *in vivo*, we injected cocktails of M23D DMD AON with myostatin or control AONs once a day for four times, with a 48 hours resting time between the second and third injections. RT-PCR analysis was performed from RNA isolated from proximal, medial and distal parts of the muscle (Figure 6B). 6 hours after the last injection, we already observed weak myostatin skip in one of the two injected muscles. At the later time points, the skipping levels modestly increased, suggesting a trend of accumulations of skipping over time. Conversely, the *Dmd *skips showed relatively more stable and more importantly higher skipping levels.

## Discussion

DMD patients suffer from progressive muscle wasting due to the absence of functional dystrophin protein. The AON-mediated exon skipping therapy has been shown to successfully reframe the mutated *DMD *gene and restore local dystrophin synthesis in DMD patients [12,13]. This approach is currently viewed as one of the most promising therapeutic approaches for DMD. However, it targets dystrophin transcripts, which are produced only in muscle and not in adipose and fibrotic tissues, thus does not specifically aim to enhance muscle regeneration or reduce fibrosis levels. To address both the underlying genetic defect and the loss of muscle, we here explored the possibility of using AONs to inhibit myostatin expression and AONs to reframe dystrophin transcripts simultaneously.

Our results show that the second exon of the *MSTN *gene can be skipped in multiple myotube cultures, either derived from healthy individuals, DMD patient or mouse. The removal of exon 2 disrupts the open reading frame and introduces premature stop codon. We observed downregulation of myostatin transcript levels up to 50-80%, depending on the type of cells tested. One can expect the truncated transcripts to be degraded via nonsense mediated decay, thus not being translated into proteins. Nonetheless, we observed a clear skip product in the RT-PCR analysis, suggesting that the transcript might be fairly stable. However, the premature stop codon introduced will result in truncated myostatin protein that lacks ~25% of the propeptide and the entire mature domains, which will abrogate its downstream signaling.

The effects of interventions with myostatin expression in muscle differentiation were assessed by increased expressions of *MYF5 *and *MYOG*. Although the expression levels of these genes were increased in all samples transfected with MSTN AON, there was no clear correlation between the fold decrease of *MSTN *and the fold increase of *MYF5 *and *MYOG*. One explanation for this observation could be that a threshold effect of myostatin knockdown on the expression of these genes is already attained when the *MSTN *transcript is slightly downregulated. However, the fact that we observed the upregulation of these genes in three to four independent experiments using different myoblasts suggests that this effect of myostatin downregulation using MSTN AON is valid and reproducible.

Finally, the mix of myostatin and dystrophin AONs showed that skipping two genes is feasible without interfering each other. The experimental set up was varied by mixing the AONs before complexing with the transfection reagent, or by preparing complex of AON and transfection reagent separately before mixing with each other to rule out the possibility that two AONs hybridize to each other. There seems to be no difference in the skipping efficiency, regardless if both AON were pre-mixed or not. However, we recommend to check the hybridization-likelihood by verifying the sequences of both AONs using freely available software tools such as RNAstructure [48].

One less encouraging finding was considerably low *Mstn *skipping level *in vivo *compared to the cultured cells. The M23D dystrophin AON, which was injected as a control in the contralateral muscle resulted in higher dystrophin skipping efficiency, confirming that the AON was injected properly. Repeated injections only showed modest improvements in the exon skipping level of myostatin. We also observed heterogenous skipping patterns throughout the muscles which seem to be specific for *Mstn *and not *Dmd*. We offer two reasons to explain the low myostatin skipping *in vivo. *First, we noted that the myostatin exon skipping efficiency in mouse cells was lower than in human cells *in vitro*. Thus, there is a possibility of species-dependent differences in the efficiency of our myostatin AON, although the AON was designed for both human and mouse. Another explanation might be that myostatin is also expressed in muscle satellite cells and fibroblasts. In vitro, we indeed saw higher *MSTN *expression in primary cultures containing mix populations of fibroblasts and myoblasts than in immortalized myoblasts. We previously demonstrated that the leaky muscle membrane facilitates the AON to enter the muscle fibers, where dystrophin is predominantly expressed, thus explaining the high efficiency of dystrophin AON. The delivery of the AON to fibrotic area and satellite cells, where myostatin is also expressed will presumably be more difficult. Furthermore, a recent study by Kang *et al *[49] which also targeted myostatin exon 2 showed efficient exon skipping *in vivo *using octa guanidine morpholino oligomers. The chosen chemistry seems to be the key to facilitate the delivery and thereby inducing prominent exon skipping *in vivo*. Finally, several studies have shown that members of the TGF-beta family other than myostatin are responsible in inhibiting muscle differentiation [35,50,51]. Therefore, as a future prospective, targeting multiple ligands seems to be a more promising strategy, especially when combined with dystrophin restoration.

## Conclusions

In conclusion, our results provide a rationale for the use of two AONs targeting two different genes and further extend the versatility of exon skipping-based therapy. The efficiency and delivery techniques remain to be improved, especially for further *in vivo *studies. While the correction of genetic defect is essential, combination with other adjuvant therapies which do not rely solely on myostatin inhibition, but also other TGF-beta family members regulating muscle differentiations might be more beneficial for DMD therapies.

## Competing interests

The authors declare that they have no competing interests.

## Authors' contributions

DUK performed experiments and wrote the manuscript, WMH participated in study design and performed experiments, SHvH and ST performed experiments, DjdG, JTdD, GJBvO, AAR and PtD participated in study design, PAtH conceived and coordinated the study. All authors read and approved the final manuscript.

## Pre-publication history

The pre-publication history for this paper can be accessed here:

http://www.biomedcentral.com/1755-8794/4/36/prepub

## Supplementary Material

Additional file 1 **Human primary control (KM109) myoblasts were differentiated for 7 days before transfection with 3 different MSTN AONs at 500 and 100 nM concentrations**. All AONs were designed to target exon 2 of the myostatin gene. The sequences are listed in table 1. RNA was isolated 2 days post-transfection. cDNA was synthesized using random hexamer (N6) primers and subjected for PCR using primers in exon 1 and 3. Click here for file
